# Microglia-Derived Small Extracellular Vesicles Reduce Glioma Growth by Modifying Tumor Cell Metabolism and Enhancing Glutamate Clearance through miR-124

**DOI:** 10.3390/cells10082066

**Published:** 2021-08-12

**Authors:** Carmela Serpe, Lucia Monaco, Michela Relucenti, Ludovica Iovino, Pietro Familiari, Ferdinando Scavizzi, Marcello Raspa, Giuseppe Familiari, Laura Civiero, Igea D’Agnano, Cristina Limatola, Myriam Catalano

**Affiliations:** 1 Department of Physiology and Pharmacology, Sapienza University, 00185 Rome, Italy; carmela.serpe@uniroma1.it (C.S.); lucia.monaco@uniroma1.it (L.M.); 2 Department of Anatomical, Histological, Forensic Medicine and Orthopedics Sciences, Sapienza University, 00185 Rome, Italy; michela.relucenti@uniroma1.it (M.R.); Giuseppe.familiari@uniroma1.it (G.F.); 3 Department of Biology, University of Padova, 35131 Padova, Italy; ludovica.iovino@unipd.it (L.I.); laura.civiero@unipd.it (L.C.); 4 Department of Human Neurosciences, Division of Neurosurgery, Sapienza University, 00185 Rome, Italy; pietro.familiari@uniroma1.it; 5 Institute of Biochemistry and Cell Biology (IBBC), CNR, 00015 Monterotond, Italy; ferdinando.scavizzi@cnr.it (F.S.); marcello.raspa@cnr.it (M.R.); 6 IRCCS San Camillo Hospital, 30126 Venice, Italy; 7 Institute of Biomedical Technologies, CNR, 20054 Segrate, Italy; igea.dagnano@cnr.it; 8 Department of Physiology and Pharmacology, Laboratory Affiliated to Istituto Pasteur Italia Fondazione Cenci Bolognetti, Sapienza University, 00185 Rome, Italy; 9 IRCCS Neuromed, 86077 Pozzilli, Italy

**Keywords:** microglia, extracellular vesicles, brain tumors, glioma, neurotoxicity, glutamate, Glt-1, miR-124

## Abstract

Brain homeostasis needs continuous exchange of intercellular information among neurons, glial cells, and immune cells, namely microglial cells. Extracellular vesicles (EVs) are active players of this process. All the cells of the body, including the brain, release at least two subtypes of EVs, the medium/large EVs (m/lEVs) and small EVs (sEVs). sEVs released by microglia play an important role in brain patrolling in physio-pathological processes. One of the most common and malignant forms of brain cancer is glioblastoma. Altered intercellular communications constitute a base for the onset and the development of the disease. In this work, we used microglia-derived sEVs to assay their effects in vitro on murine glioma cells and in vivo in a glioma model on C57BL6/N mice. Our findings indicated that sEVs carry messages to cancer cells that modify glioma cell metabolism, reducing lactate, nitric oxide (NO), and glutamate (Glu) release. sEVs affect Glu homeostasis, increasing the expression of Glu transporter Glt-1 on astrocytes. We demonstrated that these effects are mediated by miR-124 contained in microglia-released sEVs. The in vivo benefit of microglia-derived sEVs results in a significantly reduced tumor mass and an increased survival of glioma-bearing mice, depending on miR-124.

## 1. Introduction

Two types of extracellular vesicles (EVs) have been described, and both kinds can be released from all the cells of the body, including the brain. They comprise large EVs (m/lEVs) or microvesicles (MVs), originating from the outward bedding of the plasma membrane and rapidly released by the cells and small extracellular vesicles (sEVs) or exosomes (Exos) generated from the inward bedding of endocytic membranes and released by regulated exocytosis. sEVs and m/lEVs also differ in size, with the size of the former ranging from 200 to 1000 nm and that of the latter from 10 to 200 nm. No specific biomarkers distinguish the two populations of EVs [1,2]. Various cells, at different functional states, release EVs with different surface and luminal cargos [1,2]. EVs present different mechanisms of recognition and internalization into target cells [3], and both m/lEVs and sEVs are engulfed, merged or recognized by a receptor-mediated process [4].

sEVs, originated in the brain or in other organs that reach the target brain region via cerebrospinal fluid or the vascular system, instruct different recipient cells of the brain microenvironment by their content that is protected from extracellular enzymatic digestion [5]. Target cells could be both normal and neoplastic ones [6], and Exo-transferred content could exert, in gliomas, onco-suppression or onco-promotion [7].

While neurons and astrocytes represent the majority of the brain population, microglia account for 5%–12% of total brain cells, depending on the cerebral region [8]. Their physiological role is maintaining the homeostasis of the central nervous system (CNS) monitoring tissue for debris and pathogens and clearing them via phagocytic activity. sEVs released by microglial cells, the brain-resident immune cell, are key regulators of brain inflammation, being important not only to spread pro-inflammatory stimuli, as shown in Alzheimer disease and ischemia [9,10], but also to reduce neuroinflammation after traumatic brain injury [11].

In the context of gliomas, we recently demonstrated that the infusion of m/lEVs derived from lipopolysaccharide/interferon-γ (LPS/IFN-γ)-treated microglia in glioma-bearing mice reduces tumor size, which is an effect due to the mRNA cargo capable to amplify the antitumor response of microglia [12].

In the biology of brain tumors, and particularly in gliomas, the importance of microglia/macrophages, which represent up to 50% of the tumor mass [13], has been extensively investigated (revision in [14,15,16]) and has revealed evidence on the initial ability of these cells to interfere with tumor progression, which soon turn into a support for tumor invasion [17,18] and growth by the release of several soluble factors such as stress-inducible protein 1 (STI1), epidermal growth factor (EGF), interleukin 6 (IL-6), and transforming growth factor-β (TGF- β) [19,20,21].

Tumor cells also release soluble factors, which in addition to attract and modify immune cells towards a tumor-supportive phenotype are proficient to modify the microenvironment allowing tumor cell growth and migration [22]. One of these factors is glutamate: tumor growth is autocrinally facilitated by the excess of glutamate released by glioma cells that have a dysfunctional glutamate metabolism [23]. In fact, glioma cells overexpress the cystine–glutamate transporter (xCT), which transports glutamate outside the cell and lowly express the excitatory amino acid transporters Glt-1 and Glast [24,25]. High extracellular levels of glutamate sustain neurotoxicity. Consequently, neuronal death creates new spaces in brain parenchyma, facilizing glioma cell migration [26]. Another factor is nitric oxide (NO), a radical molecule, which is important for many pathophysiological processes and synthesized in high concentration by the inducible isoform of nitric oxide synthase (iNOS) from l-arginine [27]. In glioma, iNOS expression increases in correlation with higher tumor grades, and iNOS represents a key player of tumor growth sustainability [28,29].

Small highly conserved non-coding microRNAs (miRNAs), of approximately 22 nucleotides, modulate the mRNA translation binding the 3′ untranslated regions of mRNAs [30]. miRNAs transfer between cells is mediated by sEVs [31], and miRNAs act as both onco-promoters and onco-suppressors in malignant glioma biology [7].

miR-124 is counted among onco-suppressors, being one of the most downregulated miRNAs in Glioblastoma Multiforme (GBM) patients compared to in the normal brain [32,33,34]. miR-124 overexpression strongly reduces GBM aggressiveness [35,36]. In BV2 cells, miR-124 upregulation reduces the expression of iNOS [37] and reduces NO release in endothelial cells [38]. Moreover, astrocytes stimulated with Exos isolated from a neuron-conditioned medium increase miR-124 expression and Glt-1 protein levels, and miR-124 transfection into astrocytes directly produces the same effect [39].

In other tumors, such as colorectal cancer, miR-124 reduces cell growth, inhibiting the glycolytic pathways [40], and in medulloblastoma [41,42] and breast cancer, miR-124 overexpression negatively regulates the expression of the proton-linked membrane carrier (*Slc16a1*), which transports monocarboxylates (i.e., lactate, pyruvate, and ketone bodies) across the cell membrane [41,43].

In this work, we revealed that microglia-derived sEVs modulate tumor growth by carrying a specific miRNA, miR-124, to astrocytes and tumor cells, modifying glioma metabolism and especially acting on glutamate homeostasis. This mechanism has been exploited in glioma-bearing mice in which the exogenous increase of microglia-derived sEVs exerts an efficacious anti-tumor effect.

## 2. Materials and Methods

### 2.1. Cell Lines

BV2 murine microglial cells, GL261 and GL261-RFP murine glioma cells were cultured in DMEM supplemented with heat-inactivated FBS (10% for BV2 or 20% for GL261 and GL261-RFP), 100 IU/mL penicillin G, 100 mg/mL streptomycin, and 2.5 mg/mL amphotericin B and grown at 37 °C in a 5% CO_2_ and humidified atmosphere. GL261-RFP cells were obtained as previously described [44].

### 2.2. Primary Microglial Cultures

Microglial cells were obtained from mixed glia cultures derived from the cerebral cortices of C57BL6/N mice at post-natal days 0–2 (P0–P2). Cortices were chopped and digested in 15 U/mL papain (Sigma-Aldrich, Cat# P3125) at 37 °C for 20 min. Cell suspensions were plated (5 × 10^5^ cells/cm^2^) on poly-L-lysine hydrobromide (0.1 mg/mL; Sigma-Aldrich, Cat# P2636)-coated flasks in a growth medium supplemented with 10% FBS. After 9–11 days, the cultures were shaken at 37 °C for 2 h to detach and collect microglia cells. These procedures gave almost pure microglial cell populations as previously described [45] and were 98% Iba1-positive.

### 2.3. Microglia Stimulation (Polarization)

BV2 cells and primary microglia were treated with IFNγ (20 ng/mL) and LPS (100 ng/mL) or with IL-4 (20 ng/mL) for 24 h.

### 2.4. Extraction of sEVs

Microglia cells were stimulated for 30 min with ATP (1 mM) in KRH (125 mM NaCl; 5 mM KCl; 1.2 mM MgSO_4_; 1.2 mM KH_2_PO_4_; 2 mM CaCl_2_; 6 mM D-glucose; 25 mM HEPES/NaOH, pH = 7.4). The cell supernatant was collected and centrifuged at 800× *g* for 5 min to remove cell debris. The obtained supernatant was centrifuged at 10,000× *g* at 4 °C for 30 s, and the supernatant underwent ultracentrifugation at 100,000× *g* at 4 °C for 1 h. The resulting pellet, containing sEVs, was resuspended in 0.22 μm filtered phosphate buffer (PBS) or in a sEVs-free fraction medium, depending on the subsequent experiments. To obtain a sEVs-free fraction medium, the conditioned media of BV2 cells was isolated and submitted to the above reported steps of centrifugation, and the supernatant was collected and used as a vehicle or control.

### 2.5. Nanoparticle Tracking Analysis (NTA)

sEV samples obtained from the 100,000× *g* centrifugation step were resuspended in PBS and analyzed using Nanosight NS300 (Malvern Panalytical, Malvern, UK). Videos were analyzed by the inbuilt NanoSight Software NTA 3.4 Dev Build 3.4.4. The camera type, camera level, and detection threshold were sCMOS, 14, and 4, respectively. The number of completed tracks in NTA measurements was 5 (a 60 s movie was registered for each measurement). The sample was diluted in 0.22 µm filtered PBS to a final volume of 1 mL. The ideal concentration was assessed by pre-testing the optimal particle per frame value (20–100 particles per frame).

### 2.6. NO Assay

Microglia or GL261 cells (2 × 10^5^ cells/mL) plated onto 24-well plates were treated with a vehicle or with BV2-derived sEVs (3 × 10^8^ sEVs/mL) for 24 h. The supernatant was then collected and assessed for NO production by using Griess reagent (Promega). Briefly, the supernatant was mixed with an equal volume of Griess reagent and then co-incubated at room temperature (RT) for an additional 10 min. Absorbance was spectrophotometrically measured at 540 nm, and NO concentration was calculated by extrapolation from a standard curve of sodium nitrite (NaNO_2_).

### 2.7. Glutamate Release

GL261 cells (2 × 10^5^ cells/mL) plated onto 24-well plates were treated with the vehicle or with BV2-derived sEVs (3 × 10^8^ sEVs/mL). After 24 h, the cell medium was collected to measure glutamate release according to the manufacturer’s instructions (MAK005, Sigma-Aldrich, St. Louis, MI, USA). The cells were lysed in RIPA buffer and protein content quantified by BCA assay (Pierce) and used for normalization.

### 2.8. Lactate Release

GL261 cells (2 × 10^5^ cells/mL) plated onto 24-well plates were treated with the vehicle or with BV2-derived sEVs (3 × 10^8^ sEVs/mL). After 24 h, the cell medium was collected to measure lactate release according to the manufacturer’s instructions (MAK064, Sigma-Aldrich). The cells were lysed in RIPA buffer. Protein content was quantified by BCA assay (Pierce) and used for normalization.

### 2.9. Cell Transfection 

BV2 and GL261 cells (2 × 10^5^ cells/mL) plated onto 24-well plates were mock-transfected or transfected with a miR-124 mimic (4464014, Life Technologies, Carlsbad, CA, USA) or a miR-124 inhibitor (4464009, Life Technologies) via Lipofectamine 3000 Reagent (L3000001, Invitrogen, Waltham, MA, USA) in Opti-MEM according with manufacturer instructions. All types of analysis were performed 48 h after transfection. Transfection efficiency was detected via qRT-PCR.

### 2.10. In Vivo Experiments

Experiments were approved by the Italian Ministry of Health (protocol number: 694/2016- PR) in accordance with the ethical guidelines on the use of animals from the EC Council Directive 2010/63/EU. All efforts were made to minimize the number of animals used and their suffering. GL261 or GL261-RFP cells were injected in the right striatal brain region of eight-week-old male C57BL/6N mice. During surgery, a guide cannula was placed 2 mm deep in the striatum, and it was fixed with quick-setting cement. After 7 and 14 days, mice were infused via cannula with sEVs obtained from 10^6^ microglia cells (3 × 10^8^ sEVs) or 10^7^ microglia cells (3 × 10^9^ sEVs) re-suspended in 4 μL PBS. Specifically, a needle was inserted into the cannula and connected to the Hamilton syringe, and the administration was made by an infusion pump at a constant speed of 0.5 μL/min. The day after the second infusion, animals were sacrificed and analyzed for tumor size [46] or for immunofluorescence. Alternatively, mice were deeply anesthetized and CD11b^+^ cells were isolated as already described [46] by magnetic cell separation (MACS, 130-126-725, Milteniy). The obtained cells were lysed in Trizol reagent (Invitrogen, Milan, Italy) for RNA extraction, quantification, and qPCR analysis. For Kaplan–Meier survival curves, GL261-bearing mice were housed, until they reached euthanasia endpoints. 

### 2.11. Tumor Size Analysis

Coronal brain sections (thickness: 20 μm) were prepared by standard procedures and stained with hematoxylin and eosin. A section every 80 μm was collected, and the tumor size was evaluated using Image Tool 3.00. A minimum of 6 mice per experimental group were used.

### 2.12. Tissue Sampling for Transmission Electron Microscopy

GL261-bearing mice were treated as above. The day after the second infusion, animals were sacrificed. Brains were isolated, and 1 mm-thick slices were made on the coronal plane at the point of GL261 cell injection by using adult mouse brain stainless steel matrices (RBMS-200C, WPI). The biopsies were promptly fixed as reported below.

### 2.13. Transmission Electron Microscopy (TEM) 

Biopsies from peritumoral and tumoral regions of glioma-bearing mice treated with BV2-derived sEVs were fixed in 2.5% glutaraldehyde in 0.1 M PBS at pH 7.4. After fixation, the samples were rinsed in 0.1 M PBS at pH 7.4, post-fixed with 1% osmium tetroxide (OsO₄) (Agar Scientific, Stansted, UK) in 0.1 M PBS at pH 7.4 and rinsed with ultrapure water (Direct Q^®^ 3UV, Merck, Milan, Italy) for 20 min. With the purpose to obtain an increased contrast and a sharp delineation of plasma membrane-bound sEVs, samples were then incubated in 1% tannic acid (Carlo Erba Reagents, Milan, Italy) in ultrapure water (Direct Q^®^ 3UV, Merck, Milan, Italy) for 30 min and successively rinsed with the same ultrapure water for 30 min [47]. The samples were then dehydrated in ascending series of ethanol (Carlo Erba Reagents, Milan, Italy), immersed in propylene oxide (BDH Italy, Milan, Italy) for solvent substitution and embedded in epoxy resin (Electron Microscopy Sciences, Hartfield, PA 19440, USA). Silver-gold ultrathin sections with about 70 nm in thickness were obtained using a Leica EM-UC6 ultramicrotome equipped with diamond knife (Diatome, Microlab Equipments). Ultrathin sections were collected on copper grids (200 Mesh, Assing Rome, Italy) and stained with 2% lead citrate (Electron Microscopy Sciences, Hatfield, PA 19440, USA) and the UranyLess staining solution (Electron Microscopy Sciences, Hatfield, PA 19440, USA); observations were carried on by a transmission electron microscope (Carl Zeiss EM10, Thornwood, NY) operating at 60/80 kV conditions and equipped with a digital camera (AMT CCD, Deben XR-80, 8 Mp, UK Ltd., Suffolk IP309QS, UK).

### 2.14. Immunofluorescence

For glial fibrillary acidic protein (GFAP), Ki-67, and BrdU staining, coronal brain sections (thickness: 20 μm) were washed in PBS, blocked (3% goat serum and 0.3% Triton X-100 in PBS) at RT for 1 h and incubated at 4 °C overnight, with the following primary antibodies: rabbit anti-GFAP (1:500, Novus, NB300-141), rabbit anti-Ki-67 (1:50, Spring, M3062), and rat anti-BrdU (1:200, Novusbio, NB500-169). The slices were washed in PBS and stained at RT for 1 h with the secondary antibodies conjugated to Alexa Fluor 488 or Alexa Fluor 568 (1:500, Life Technologies) used as appropriate. Hoechst (stained at RT for 1 h; 1:1000 Molecular Probes, 33342) was used for nuclei visualization. The slides were mounted with Dako Fluorescence Mounting Medium (S3023). Analysis was performed using a fluorescence microscope (Nikon Eclipse Ti) by MetaMorph 7.6.5.0, measuring the signal coverage area of GFAP, Ki-67, and BrdU normalized by tumor area upon setting the scale and the threshold. For Glt-1, Glast, and glutamine synthetase (GS) staining, coronal brain slices (thickness: 20 μm) were washed in PBS. To quench intrinsic autofluorescence, the slices were incubated with 50 mM NH_4_Cl in PBS for 15 min. Subsequently, the slices were washed in PBS and saturated at RT for 1 h in a blocking solution (1% bovine serum albumin (BSA), 15% goat serum, 0.25% gelatin, 0.20% glycine, and 0.5% Triton). Then, the slices were incubated at 4 °C overnight, with the following primary antibodies: guinea pig anti-Glt-1 (1:400, AB1783, EMD Millipore), rabbit anti-GS (1:300, GTX109121, GENETEX), or anti-Glast (1:250, 134060, GeneTex). The slices were washed in PBS and incubated at RT for 1 h with the secondary antibodies conjugated to Alexa Flour 488, Alexa Fluor 568, or Alexa Fluor 633 fluorophores (1:200; Life Technologies) used as appropriate. Hoechst was used for nuclei visualization. The slices were mounted with Mowiol (Calbiochem). Analysis was performed using the Leica SP5 confocal microscope with an HC PL FLUOTAR 40 × /0.70 oil objective at an 8-bit intensity resolution over 1024 pixel × 1024 pixel. We acquired 5 images per each analyzed slice. Using ImageJ, we measured the integrated density (IntDen, calculated as area × mean fluorescence) of Glt-1, GS, and Glast signal upon setting the scale and the threshold. 

### 2.15. BrdU Proliferation Assay

GL261 cells were grown on glass coverslips (2 × 10^5^ cells/mL) in 24-well plates for 18 h. Cells were exposed to a vehicle or sEVs for 24 h, and cellular proliferation was analyzed adding BrdU (10 mM; Sigma-Aldrich, B5002) for 30 min. The cells were washed in PBS, fixed (4% PFA; duration: 30 min), permeabilized (1% Triton X-100; duration: 15 min), blocked (1% BSA; duration: 1 h) at RT and incubated overnight with anti-BrdU (1:200; Novusbio, NB500-169). Hoechst was used to stain all nuclei. Proliferation rates were calculated as BrdU^+^ cells with respect to Hoechst-stained cells. Fifteen days after glioma cell injection, BrdU was intraperitoneal injected (50 mg/kg). Two hours later, the mice were killed, and their brains processed for immunofluorescence as above.

### 2.16. qRT-PCR

Total RNAs were extracted from cells or from sEVs with Trizol reagent (T9424, Sigma, St. Louis, MO, USA) or Total EXO RNA Extraction Kit (4478545, Sigma) according to the manufacturer’s instructions [48]. RNAs extracted from all samples were quantified and reverse-transcribed using IScriptTM Reverse Transcription Supermix (1708841, Bio-rad, Hercules, CA, USA) or TaqMan MicroRNA Reverse Transcription Kit (4366596, ThermoFisher, Waltham, MA, USA) according to the manufacturer’s description. qRT-PCR was carried out in an I-Cycler IQ Multicolor RT- PCR Detection System (Bio-rad) using SsoAdvanced Universal SYBR Green Supermix (1725274, Bio-rad) or with TaqMan MicroRNA Assay kit (4427975, ThermoFisher) according to the manufacturer’s protocol. The PCR protocol consisted of 40 cycles of denaturation at 95 °C for 30 s and annealing/extension at 58 °C for 30 s. Equal amounts of RNAs of all samples (cells and sEVs) were analyzed. For quantification analysis, the comparative threshold cycle (Ct) method was used. The Ct values from each gene were normalized to the Ct value of *Gapdh* or U6 in the same RNA sample (ΔCt). Relative quantification was performed using the 2^−ΔΔCt^ method and expressed as fold increase. Primer sequences for mRNA analysis are listed in Table 1.

### 2.17. NanoString nCounter Analysis

Total RNAs were isolated from microglia-derived sEVs by Total Exosome RNA Protein Isolation Kit (# 4478545, Invitrogen, Waltham, MA, USA) and concentrated using the Microcon10 centrifugal filters (#MRCPRT010, Merck Millipore). Raw Gene expression data were normalized considering *Gapdh* as a housekeeping gene.

### 2.18. Statistical Analysis

All data were analyzed using SigmaPlot 11.0 Software. Distributions of survival times were described using the Kaplan–Meier method. All tests were two-sided considering an overall significance level of 0.05. Between-group differences were analyzed by comparison statistical tests reported for each experiment in the consistent figure legend.

## 3. Results

### 3.1. Microglia-Derived sEVs Exert Anti-Tumoral Activity in Glioma-Bearing Mice Independent of the Inflammatory State of Donor Cells

**Figure 1:**
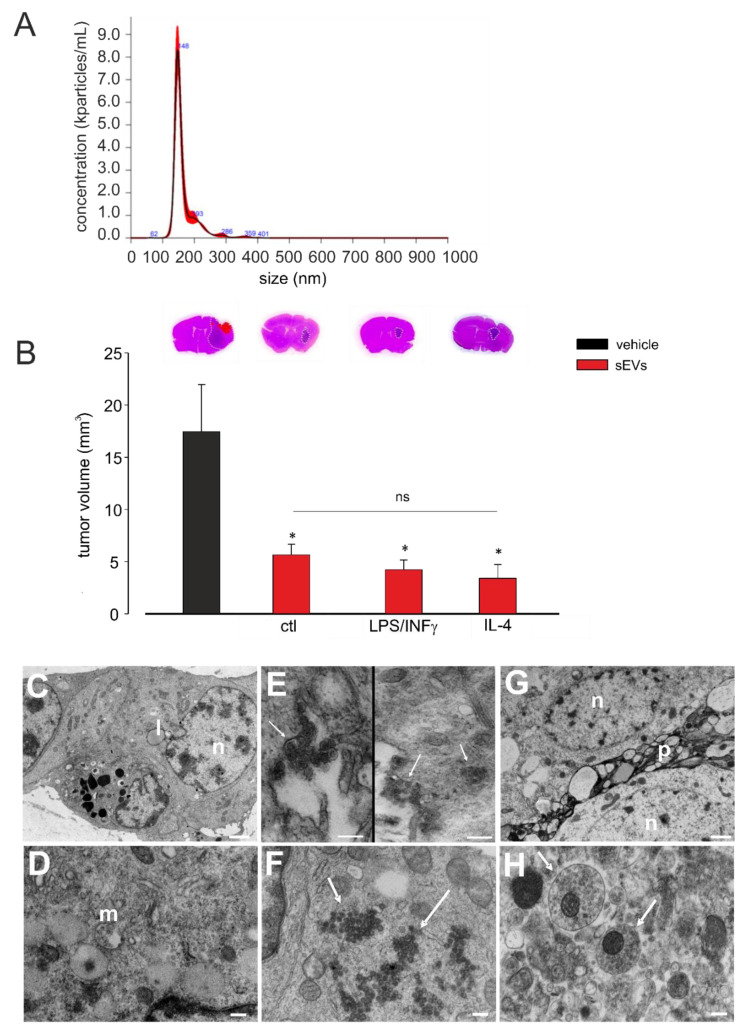
Size and concentration analysis of microglia-derived small extracellular vesicles (sEVs), their effects in glioma-bearing mice and their morphological analysis. (**A**) Representative concentration/size graph from the nanoparticle tracking analysis (NTA) of BV2-derived sEVs. The mean concentration (±standard error, SE; in particles/mL) and mean size (±SE; in nm) of sEVs, obtained from five independent experiments, are reported in the main text. (**B**) Tumor size in the brain of GL261-bearing mice treated with an sEVs-free fraction isolated from the cell medium of BV2 cells (vehicle) and sEVs isolated from the cell medium of untreated (ctl), lipopolysaccharide/interferon-γ (LPS/IFNγ)-treated, or interleukin 4 (IL-4)-treated BV2 cells. Tumor size (in mm^3^) was reported as mean ± SE; N = 7/experimental group; * *p* < 0.05, vs. vehicle, by using Student’s *t*-test; *p* = 0.17 among sEVs treatments, by using the Kruskal–Wallis ANOVA test. On the top, representative coronal brain sections of GL261-bearing mice were treated as above and stained with hematoxylin and eosin; the tumor area is indicated by the white dashed line. (**C**–**H**) Representative transmission electron microscopy (TEM) images of the brain biopsies of GL261-bearing mice treated with BV2-deribed sEVs. In (**C**), glioma cells showed irregular nuclei (n), prominent nucleoli, and lipid droplets (l) (magnification: 14.000×; scale bar: 1 µm). In (**D**), glioma cells showed endoplasmic reticulum and multivesicular bodies (m) (magnification: 56.300×; scale bar: 200 nm). In (**E**), on the left side, glioma cells showed membrane-bearing sEVs (indicated by arrows; magnification: 73.400×; scale bar: 200 nm); on the right side, glioma cells showed the macropinocytosis and internalization of sEVs (indicated by arrows; magnification: 44.900×; scale bar: 400 nm). (**F**) sEVs in glioma cells cytoplasm (indicated by arrows; magnification: 56.300×; scale bar: 200 nm). (**G**) Normal astrocytes with oval nuclei (n), dispersed chromatin, and cytoplasmatic organelles and processes (*p*) (magnification: 13,400×; scale bar: 1 µm). (**H**) sEVs in astrocyte processes (indicated by arrows; magnification: 56.300×; scale bar: 200 nm).

sEVs were obtained from the BV2 microglial cell line. The NTA performed to determine the size and the number of the sEVs isolated from BV2 cells showed that the mean size was below 200 nm (191.2 ± 20.9 nm; N = 5) and the sEV mean concentration was 12.9 × 10^3^ (±2.6 × 10^3^) nanoparticles/mL (Figure 1A).

Adult male mice were injected with GL261 glioma mouse cells in the striatal brain region. After 7 and 14 days, the mice were infused (as reported in Method Section) with 3.0 × 10^8^ (±1.2 × 10^7^) sEVs, collected from untreated, LPS/IFNγ-treated, or IL-4-treated BV2 cells. As a control, an sEVs-free fraction was isolated from the conditioned media of BV2 cells and administered to tumor-bearing mice. Seventeen days after tumor injection, tumor volume was evaluated by the hematoxilin/eosin staining of coronal brain sections, and as shown in Figure 1B, all treatments significantly reduced the tumor volume and at the same extent, indicating an effect independent of the inflammatory status of the donor microglial cells. The phenotype of donor cells after IL-4 or LPS/IFNγ treatment was confirmed by mRNA analysis and functional assays, as previously reported [12,46]. Moreover, the mRNA contents of sEVs released by untreated, LPS/IFNγ-treated, or IL 4-treated microglia were analyzed by NanoString chip for the expression of 243 key inflammation-related genes. Eleven genes were found to be upregulated in sEVs derived from LPS/IFNγ-treated microglia, specifically *Ccl2*, *Ccl3*, *Ccl5*, *Ccl7*, *Cd7*, *Cebpb*, *Cxcl9*, *Hif1a*, *Il1b*, *Rapgef2*, and *Tnf-α*, while *Ccl3*, *Il1b*, *Tnf-α*, and *Rapgef2* were downregulated in sEVs derived from IL-4-treated microglia (all compared to in sEVs released by untreated microglia; Table S1). The data of the immune system panel were validated by qRT-PCR: *Il1b* and *Tnf-α* genes were significantly upregulated in sEVs released by LPS/IFNγ-treated microglia and significantly downregulated in sEVs released by IL4-treated microglia, with respect to sEVs released by untreated cells (Figure S1A,B). These findings demonstrated that, although sEVs differ in their mRNA inflammatory gene content, this difference is not a determinant of their antitumor effect.

Cerebral slices of GL261-bearing mice treated with BV2-derived sEVs were analyzed by TEM. The analysis of the tumor brain region, as represented in Figure 1C,D, showed that GL261 glioma cells appeared as closely packed and pleomorphic large cells; abundant intercellular spaces were also observed. The glioma cells showed irregular and lobulated nuclei, containing irregularly dispersed and marginated chromatin; prominent nucleoli were also observed. Cytoplasm was abundant and filled by smooth and rough endoplasmic reticulum, numerous small mitochondria, free polyribosomes, small bundles, thin filaments, and Golgi apparatus. Multivesicular bodies, electron-dense autophagic vacuoles, and lipid droplets were also observed. In the same region, sEVs appeared as lipid bilayer vesicles having a round-shape morphology and a size under 200 nm, confirming the NTA. They were observed in the intercellular spaces between tumor cells, forming small clusters adhering to the plasma membranes of glioma cells (Figure 1E, left). Some areas of glioma cell plasma membranes showed at least two different cellular uptake pathways, caveolae mediated endocytosis (Figure 1E, left) and macro-pinocytosis (Figure 1E, right). In fact, as shown in Figure 1E (right), macro-pinocytosis was confirmed by finding endosomes in the cytoplasm area beneath the plasma membrane, and it was confirmed by the occurrence of vacuoles containing sEVs in the same cellular position. Examining cytoplasm matrix content, large clusters of free vesicles escaping endo-lysosome degradation were observed, but no sEVs appeared as transiting through the nuclear membrane (Figure 1F). Outside the tumoral core, phenotypically different cells were observed with respect to glioma cells; mainly, astrocytes revealed a round or oval nucleus with dispersed chromatin and a clear cytoplasm containing mitochondria, scattered clusters of ribosomes, endoplasmic reticulum and Golgi apparatus, few microfilaments, and microtubules [49]. Numerous broad processes surrounded the cells, displaying mitochondria and more microfilaments and microtubules inside it. A great number of sEVs were observed inside the processes of these cells. To a lesser extent, sEVs was observed into their cytoplasm (Figure 1G,H). These analyses revealed that exogenous sEVs injected in the glioma-bearing mice were pinocyted not only by tumor cells, but also by astrocytes in the peritumoral area.

### 3.2. Microglia-Derived sEVs Reduce Astrocytosis in Glioma-Bearing Mice

**Figure 2:**
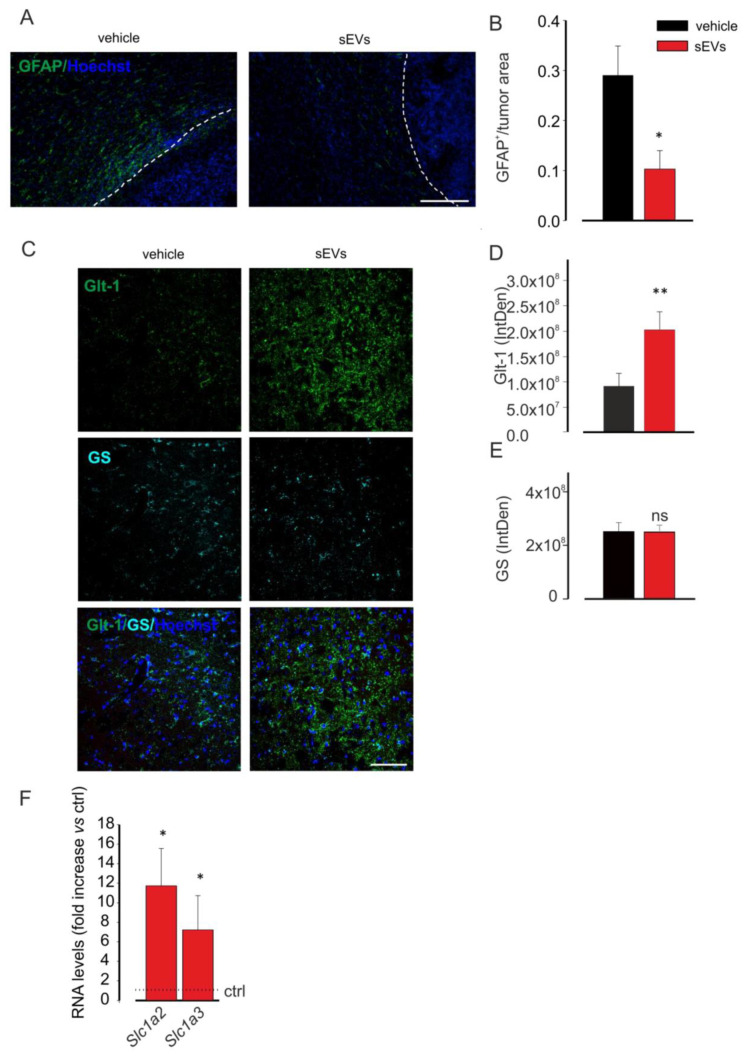
Microglia-derived sEVs effects on astrocytes. (**A**) Representative immunofluorescence analysis for glial fibrillary acidic protein (GFAP in green; Hoechst in blue) of coronal brain sections of GL261-bearing mice vehicle treated (on the left) or treated with BV2-derived sEVs (on the right). The tumor region is on the right of the dashed line. Scale bar: 20 μm. (**B**) Quantification of (**A**). Data are expressed as the signal coverage area of GFAP normalized by tumor area (GFAP^+^/tumor area ± SE; N = 3; * *p* ≤ 0.05; by using Student’s *t*-test). (**C**) Representative immunofluorescence analysis for Glt-1 (in green) and glutamine synthetase (GS in light blue; Hoechst in blue) of coronal brain sections of GL261-bearing vehicle treated or treated with BV2-derived sEVs in the peritumoral area. Scale bar: 20 μm. (**D**,**E**) Quantification of (**C**). Data are expressed as mean intensity (IntDen) ± SE; N = 3; * *p* ≤ 0.05; ** *p* ≤ 0.001; Student’s *t*-test. (**F**) qPCR of *Slc1a2* and *Slc1a3* genes in primary astrocyte cultures which were untreated (ctrl) or treated with sEVs. Data are the mean ± SE of fold increase vs. ctrl, normalized on *Gapdh* (used as a housekeeping gene); N = 6; * *p* < 0.05 vs. ctrl; by using the Mann–Whitney rank sum test.

The sEVs inoculated in the region of the tumor mass were visible also in the processes of cells into the peritumoral region and far from the tumor mass in cells that appeared phenotypically different from those in the tumor core (as shown in Figure 1G,H) [50]. Among these cells, reactive astrocytes have been described as enhancers of tumor colonization [51,52,53,54]. To investigate if untreated microglia-derived sEVs were administered to glioma-bearing mice and also affected astrocytes, we analyzed astrocyte reactivity as GFAP staining by immunofluorescence. The data shown in Figure 2A,B demonstrated that on coronal brain sections of glioma-bearing mice treated with sEVs, a significant reduction of GFAP staining was observed, indicating a reduction of astrogliosis in the peritumoral brain region.

Moreover, to identify a possible functional effect of sEVs on astrocytes, Glt-1 expression was analyzed in the peritumoral region: the data reported in Figure 2C–E indicated that the glutamate transporter was overexpressed in sEVs-treated mice, without variation in the number of astrocytes, identified as GS-positive cells.

To further confirm these data, primary astrocyte cultures were stimulated for 24 h with sEVs derived from BV2 and then analyzed by qRT-PCR for the expression of Glt-1 (*Slc1a2*) and Glast (*Slc1a3*) genes. Figure 2F shows that sEVs treatment increased the expression levels of both genes in comparison with untreated astrocytes.

The antitumor effect of m/lEVs infused into glioma-bearing mice has been attributed to the vesicle ability to modify the phenotype of tumor-infiltrating immunosuppressive cells, particularly tumor-associated macrophages/microglia (TAMs) that reacquire a homeostatic protective phenotype [12]. To investigate the effect of sEVs derived from untreated microglia on TAM inflammatory status in glioma-bearing mice, we isolated CD11b + cells from the brain of these animals. As previously shown [46], anti-inflammation-related genes (*Arg1*, *Fizz1*, and *Ym1*) were upregulated in the tumor-bearing brain hemisphere. sEV treatment only reduced the expression of *Arg1* and had no effects on *Cd163*, *Cd206*, *Fizz1*, and *Ym1* expression, indicating that sEVs do not modulate TAM phenotype (Figure S1C).

To further confirm this finding, we performed in vitro experiments on primary microglia. Cells were stimulated with a glioma-conditioned medium (gcm) and incubated with BV2-derived sEVs for 24 h. sEVs treatment did not modify the expression levels of some pro-tumor genes (*Cd163*, *Cd206*, and *Fizz-1*), which were upregulated by gcm treatment (Figure S1D). The result was also confirmed on IL-4-stimulated microglial cells: BV2-derived sEVs were not able to modify the expression of pro-tumor genes (*Arg-1*, *Cd163*, *Cd206*, *Fizz-1*, and *Ym1*), with all of them upregulated by IL-4 treatment compared to the control cells (Figure S1E).

### 3.3. Microglia-Derived sEVs Exert an Anti-Tumor Effect and Reduce Glioma Cell Proliferation in Mice Depending on the Number

**Figure 3:**
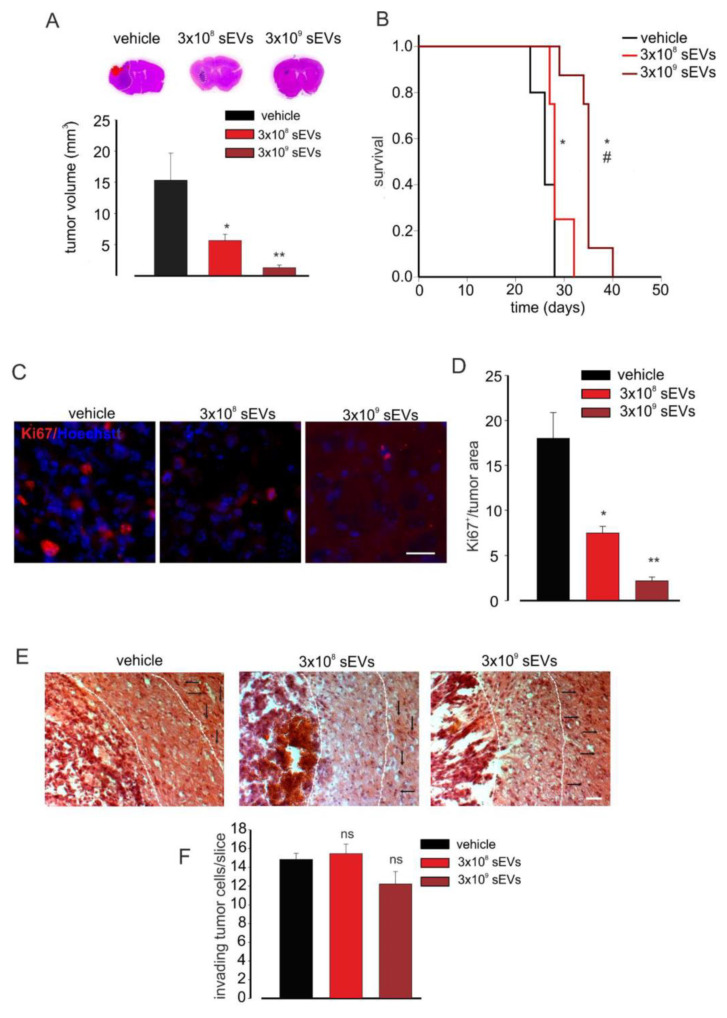
Microglia-derived sEV effects on tumor size and survival in glioma-bearing mice. (**A**) Tumor size in the brain of GL261-bearing mice treated with a vehicle or 3 × 10^8^ or 3 × 10^9^ BV2-derived sEVs. Tumor size (in mm^3^) was reported as mean ± SE; N = 7 mice/experimental group in vehicle and 3 × 10^8^ groups and N = 6 mice/experimental group in 3 × 10^9^ group; * *p* < 0.05, ** *p* ≤ 0.001 vs. vehicle; by using Student’s *t*-test. On the top, representative coronal brain sections of GL261-bearing mice treated as above, stained with hematoxylin and eosin; the tumor area is indicated by the dashed line. (**B**) Kaplan–Meier survival curves of GL261-bearing mice treated with 3 × 10^8^ or 3 × 10^9^ BV2-derived sEVs obtained from 1 × 10^6^ or 1 × 10^7^ cells. N = 10 mice/experimental group in vehicle group and N = 8 mice/experimental group in 3 × 10^9^ and 3 × 10^8^ groups; Gehan-Breslow statistic for the survival curves (*p* < 0.001); multiple comparisons: * *p* ≤ 0.05 vs. vehicle, # *p* ≤ 0.05 3 × 10^8^ sEVs vs. 3 × 10^9^ sEVs; by using the Holm-Sidak test. (**C**) Representative immunofluorescence analysis for Ki67 (in red; Hoechst in blue) of coronal brain sections of GL261-bearing treated with a vehicle and 3 × 10^8^ and 3 × 10^9^ BV2-derived sEVs. Scale bar: 50 μm. (**D**) Quantification of (**C**). Data are expressed as mean intensity (Int) per tumor area ± SE; N = 3; * *p* ≤ 0.05, ** *p* ≤ 0.001 vs. vehicle; by using the Mann–Whitney test. (**E**) Representative brain peritumoral sections stained with haematoxylin/eosin of GL261-bearing mice treated with vehicle and 3 × 10^8^ and 3 × 10^9^ BV2-derived sEVs; white arrows indicate glioma cells invading the brain parenchyma beyond the main tumour border for more than 150 μm (dashed line); scale bars: 40 μm. (**F**) Quantification of (**E**). Data are expressed as mean cell number ± SE; N = 3/experimental group; ns *p* > 0.05 vs. vehicle; by using Student’s *t*-test.

To investigate a possible effect of sEVs depending on the number, glioma-bearing mice were treated with different amounts of BV2-derived vesicles. As reported in Figure 3A, increasing amounts of sEVs had stronger effects on tumor volume reduction, as well as on animal survival time (see the Kaplan–Meier curves in Figure 3B).

We then asked if sEV treatment reduced tumor growth by affecting tumor cell proliferation. To answer this question, coronal brain sections of glioma-bearing mice treated with 3 × 10^8^ or 3 × 10^9^ sEVs derived from BV2 cells were analyzed for the expression of the proliferation marker Ki67 by immunofluorescence: the data reported in Figure 3C,D showed a significant reduction of Ki-67 staining upon treatment with both 3 × 10^8^ and 3 × 10^9^ sEVs (derived from 10^6^ and 10^7^ BV2), and the numbers of proliferating cells were significantly different between the two treatments. To ascertain the direct effect of microglia-derived sEVs on GL261 proliferation, we performed in vitro experiments: GL261 cells were treated with sEVs derived from BV2 cells (number ratio: 1:5) for 24, 48, 72, and 96 h and stained with BrdU [55]. As shown in Figure S2A,B, glioma cell proliferation (assayed by BrdU) was significantly reduced upon 48, 72, and 96 h of sEVs stimulation, indicating the direct effect of microglia-derived vesicles on glioma. To investigate the effects of sEV on glioma cell proliferation in vivo, mice were treated with BrdU (two hours before euthanasia). Figure S2C,D shows that BrdU^+^ cells, normalized by tumor area, were significantly reduced in the tumoral region in sEVs-treated mice, confirming the anti-proliferative effects on microglial sEVs. One of the main obstacles to the complete surgical resection of gliomas and to the efficacy of chemo and radiotherapy is the high ability of glioma cells to invade the surrounding parenchyma [56]. To verify if sEVs affect glioma cell movement in vivo, the tumor cell invasion of brain parenchyma was investigated by measuring the number of cells protruding more than 150 μm from the main tumor mass. The data reported in Figure 3E,F demonstrated no significant reduction of this value. Similar results were obtained with a higher amount of sEVs, confirming previous in vitro results [12].

### 3.4. Microglia-Derived sEVs Reduce the Toxicity of Glioma Cells

In the peritumoral tissue, the excitatory amino acid neurotransmitter Glu released by glioma cells can reach excitotoxic concentrations, promoting neurotoxicity [27,56,57,58,59] and enhancing the proliferation of glioma cells [60].

**Figure 4:**
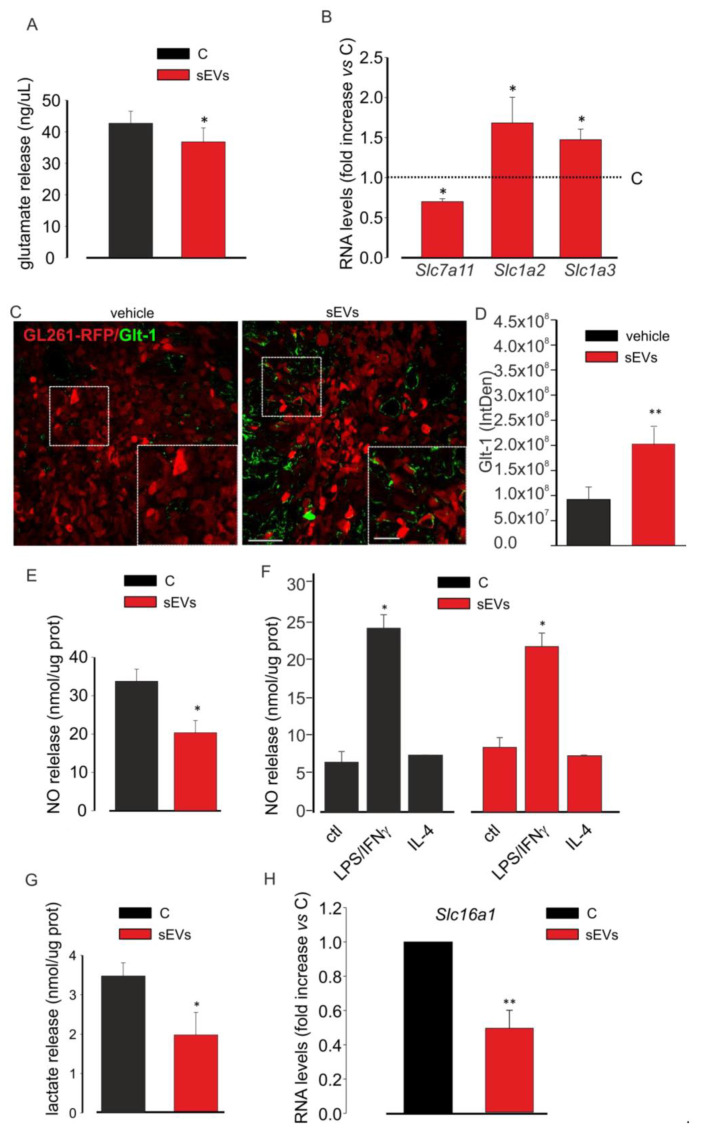
Microglia-derived sEV effects on the toxicity of glioma cells. (**A**) Glutamate release in a medium of untreated (C) or BV2-derived sEVs-treated GL261 cells. Data are expressed as ng/mL; N = 3; * *p* < 0.05 vs. C; by using Student’s *t*-test. (**B**) qPCR of *Slc7a11*, *Slc1a2*, and *Slc1a3* genes expressed in untreated (C) or BV2-derived sEVs-treated GL261 cells. Data are the mean ± SE of fold increase vs. C; normalized on *Gapdh* (used as a housekeeping gene); N = 6; * *p* < 0.05 vs. C; by using the Mann–Whitney rank sum test. (**C**) Representative immunofluorescence analysis for Glt-1 (in green; GL261-RFP in red) in the tumor core of coronal brain sections of GL261-bearing mice which were not treated (vehicle, on the left) or treated with BV2-derived sEVs (on the right); scale bars: 20 μm. In the inset, an enlargement is shown; scale bars: 50 μm. (**D**) Quantification of (**C**). Data are expressed as mean IntDen ± SE; N = 3; ** *p* ≤ 0.001 vs. vehicle; by using Student’s *t*-test. (**E**) NO release in the medium of untreated (C) or BV2-derived sEVs-treated GL261 cells. Data are expressed as nmol/mg prot; N = 3; * *p* < 0.05 vs. C; by using Student’s *t*-test. (**F**) NO release in the medium of untreated (ctl), LPS/IFNγ-treated, and IL 4-treated BV2 cells in absence (C) or in the presence of BV2-derived sEVs. Data are expressed as nmol/mg prot; N = 3; * *p* < 0.05 vs. ctl both in C and in sEVs; by using Student’s *t*-test. (**G**) Lactate release in the medium of untreated (C) or BV2-derived sEVs-treated GL261 cells. Data are expressed as nmol/mg prot; N = 4; * *p* < 0.05 vs. C, Student’s *t*-test. (**H**) qPCR of *Slc16a1* gene expressed in untreated (C) or BV2-derived sEVs-treated GL261 cells. Data are the mean ± SE of fold increase vs. C, normalized on *Gapdh* (used as a housekeeping gene); N = 8; ** *p* ≤ 0.001; by using Student’s *t*-test.

These findings prompted us to study a possible modulation of microglia-derived sEVs on the release of neurotoxic and/or pro-tumoral molecules by glioma. Therefore, assuming a lower tumorigenicity due to a lower release of these substances, we first investigated Glu release. As shown in Figure 4A, the stimulation of GL261 cells with microglia-derived sEVs reduced Glu in the cell medium.

The modulation of Glu release could be mediated by the different expression of the glutamate transport apparatus, which includes the Na^+^-independent system x_c_^−^ cystine/glutamate transporter mainly in charge of releasing glutamate and the Na^+^-dependent glutamate transporters, Glt-1 and Glast, responsible for neurotransmitter uptake. In glioma cells, the cystine–glutamate exchanger is overexpressed, while Glt1 and Glast are expressed in low extent or mislocated with a prevalent nuclear expression [24], thus causing an excess of extracellular glutamate. To verify the involvement of this apparatus in the antitumor effect of microglia-derived sEVs, GL261 cells were analyzed for the expression of these transporters by qRT-PCR. The data reported in Figure 4B demonstrated that the expression of the x_c_^−^ cystine/glutamate transporter gene (*Slc7a11*) was significantly reduced in the treated cells compared to in the control cells; in contrast, the expression levels of Glt-1 gene (*Slc1a2*) and Glast gene (*Slc1a3*) were increased upon microglia-derived sEVs stimulation. These data were also confirmed in vivo: glioma-bearing mice (inoculated with GL261-RFP cells) were treated with BV2-derived sEVs, as previously described. The reduction of tumor size upon sEVs treatment was confirmed as RFP signal quantification (Figure S3A). The brain coronal sections of vehicle- and sEVs-treated glioma-bearing mice were assayed by IF for Glt-1, and as reported in Figure 4C,D, sEVs-treated mice showed an increased expression of the transporter on the RFP-positive cells compared to that on the vehicle-treated mice. In the same brain slices, Glast expression assayed by IF was significantly increased in the sEVs-treated animals (Figure S3B,C).

Another neurotoxic and pro-tumoral molecule released by cancer cells is NO, which induces genotoxic mechanisms, promotes angiogenesis, limits host immune response against tumor and promotes metastasis [28]. For that reason, we analyzed the concentration of NO released by GL261 cells which were untreated or treated with microglia-derived sEVs: we observed a significant reduction of NO concentration in the cell medium after sEVs treatment (Figure 4E). NO is also released by pro-inflammatory microglia and could be detrimental for tumors [61]. For that reason, to verify whether sEVs could modify NO release from microglial cells, control, LPS/IFNγ-simulated, and IL-4-stimulated microglia were treated with sEVs. Figure 4F shows that these treatments did not affect NO release by LPS/IFNγ-stimulated BV2 cells (Figure 4F).

Glioma cells, as other cancer cells, undergo aerobic glycolysis using the product of this process for their vitality. In fact, lactate serves to acidify the microenvironment and to ease tumor cell proliferation and invasion; the NADH serves as a biomass fuel [62]. For these reasons, we analyzed the release of lactate by GL261 cells which were treated or not with microglia-derived sEVs and observed a significant reduction of its concentration in the glioma cell medium (Figure 4G). To confirm these data, we tested the expression of the monocarboxylate transporter 1 (MCT1 or solute carrier family 16 member 1 (*Slc16a1*)) that has been already demonstrated to provide metabolic fuels to cancer cells [63]. Figure 4H shows that the sEVs-treated GL261 cells expressed *Slc16a1* mRNAs at levels lower than the control cells.

### 3.5. miR-124 Transferred from Microglia to Glioma Cells via sEVs Is a Key Regulator of the Anti-Tumoral Effect of Microglia-Derived sEVs

**Figure 5:**
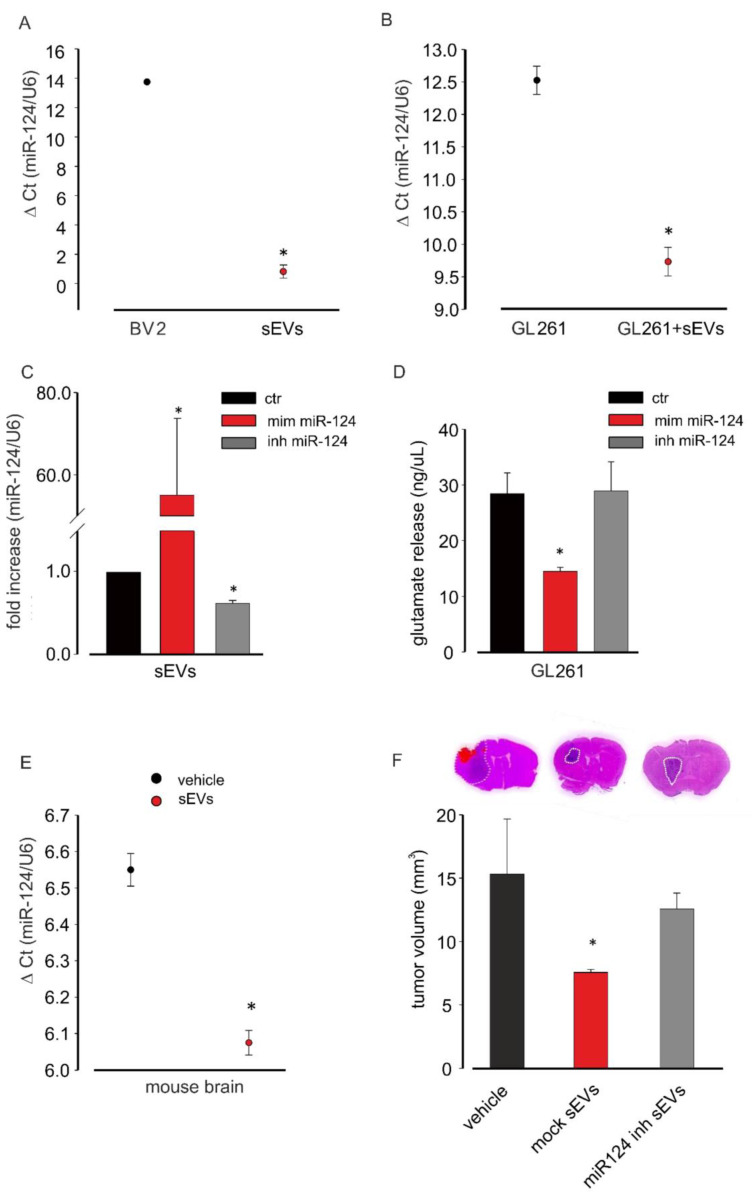
miR-124 analysis in microglia-derived sEVs. (**A**) miR-124 expression levels in BV2 cells and in BV2-derived sEVs evaluated by qRT-PCR analysis. Data are expressed as the mean ± SE of ΔCt, normalized on *u6* (used as a housekeeping gene); N = 3; each experiment was performed in duplicate; * p < 0.05; by using Student’s *t*-test. (**B**) miR-124 expression in untreated GL261 and in BV2-derived sEVs-treated GL261 evaluated by qRT-PCR analysis. Data are expressed as the mean ± SE of ΔCt, normalized on *u6* (used as a housekeeping gene); N = 3; each experiment was performed in duplicate; * p < 0.05; by using Student’s *t*-test. (**C**) miR-124 expression in sEVs derived from BV2 transfected with the vehicle (ctr), the miR-124 mimic (mim miR-124), and the miR-124-inhibitor (inh miR-124) by qRT-PCR analysis. Data are expressed as the mean ± SE of fold increase normalized on *u6* (used as a housekeeping gene); N = 3; each experiment was performed in duplicate; * p < 0.05 vs. ctr; by using Student’s *t*-test. (**D**) Glutamate release in the medium of BV2 transfected with the vehicle (ctr), the miR-124 mimic (mim miR-124), and the miR-124-inhibitor (inh miR-124). Data are expressed as ng/mL; N = 3; each experiment was performed in triplicate; * p < 0.05 vs. ctr; by using Student’s *t*-test. (**E**) miR-124 expression in the total brain extracts of GL261-bearing-mice treated with vehicle or BV2-derived sEVs evaluated by qRT-PCR analysis. Data are expressed as the mean ± SE of ΔCt, normalized on *u6* (used as a housekeeping gene); N = 3; each experiment was performed in duplicate; * p < 0.05; by using Student’s *t*-test. (**F**) Tumor size in the brain of GL261-bearing mice treated with the vehicle and sEVs derived from mock-transfected (mock sEVs) or miR-124-inhibitor-transfected (miR-124 inh sEVs) BV2 cells. Tumor size (in mm^3^) was reported as mean ± SE; N = 7 mice for vehicle and miR-124 inh sEVs experimental groups; N = 6 mice for mock sEVs experimental group; * p < 0.05; by using Student’s *t*-test. The top three figures of (**F**) show representative coronal brain sections of GL261-bearing mice treated as above and stained with hematoxylin-eosin; the tumor area is shown by the dashed line.

To investigate the hypothesis that the anti-tumor effects induced by untreated microglia-derived sEVs was in part mediated by miR-124, we first analyzed its expression in sEVs. The data reported in Figure 5A showed that sEVs were enriched in miR-124 in comparison with in BV2 donor cells and that GL261 cell treatment with sEVs increased their miR-124 expression (Figure 5B).

To verify the involvement of miR-124 in sEVs-mediated effects, we took advantage of an miR-124 inhibitor that specifically binds and inhibits endogenous miR-124. sEVs were isolated from BV2 cells transfected with the miR-124 inhibitor, and mRNAs were extracted and analyzed by qRT-PCR: Figure 5C shows a significant reduction in the expression of miR-124 after miR-124 inhibitor transfection. As a control, BV2 cells were transfected with a miR-124 mimic, and sEVs released by these cells contained a significantly increased expression of miR-124 compared to that under control conditions. To investigate a functional effect of miR-124 on glutamate machinery in GL261, cells were transfected with the miR-124 mimic or miR-124 inhibitor and assayed for the release of Glu. As shown in Figure 5D, the miR-124 mimic reduced, while the miR-124 inhibitor did not affect the release of Glu in the GL261-conditioned medium, indicating results similar to those induced by BV2-derived sEVs stimulation of glioma cells (Figure 4A).

In the in vivo glioma model, miR-124 was overexpressed in the total brain mRNA extracts of sEVs-treated mice compared to in vehicle-treated animals (Figure 5E).

To confirm in vivo the involvement of miR-124 in sEVs-mediated anti-tumor effects, we treated GL261-bearing mice with sEVs released by mock-transfected and miR-124 inhibitor-transfected BV2 cells. We again observed that sEVs derived from mocked-transfected cells reduced the tumor volume while no variations were obtained by injecting sEVs derived from miR-124-inhibitor-transfected cells (Figure 5F), confirming also in vivo that miR-124 mediates the antitumoral effect of microglia-derived sEVs.

## 4. Discussion

In this study, we demonstrated that microglia-derived sEVs exert an anti-tumor effect in glioma-bearing mice, the effect increases with the number of infused vesicles, and it is independent of the inflammatory status of donor cells. The antitumoral activity was explained by the ability of sEVs to reduce the release of Glu by tumor cells through the transfer of miR-124 that increases the expression of the Glu transporters on GL261 cells and inhibit the expression of the cystine/glutamate antiporter system x_c_^−^. As demonstrated in an in vivo model, this mechanism protects neurons from the Glu-induced neurotoxicity mediated by glioma cells [24]. Different from normal astrocytes, glioma cells have a unique expression of the Glu uptake/release system with a reduced presence of transporters (particularly Glt-1) and a high expression of the x_c_^−^ system that favors the net accumulation of Glu into the extracellular milieu. With the increased availability of Glu, the antioxidant glutathione (GSH) levels also increase, to better protect tumor cells from reactive nitrogen and oxygen species (RNS and ROS), which are produced in excess by cancer cells [64]. We observed that sEVs determined a significant decrease of x_c_^−^ expression by glioma cells that can justify the decreased accumulation of Glu in a glioma-conditioned medium. In addition, sEVs treatment increased Glt-1 expression in astrocytes in the peritumoral area and increased Glast in in vitro cultures, thus contributing to increasing the uptake of Glu and reducing glioma-induced neurotoxicity.

We reported that sEVs administration to glioma-bearing mice modulates the tumor microenvironment as a consequence of the reduction of neurotoxic molecules released by tumor cells. In fact, sEVs-treated GL261 cells release less NO than untreated cells. This NO undergoes oxidative/reductive reactions, forming toxic compounds (i.e., RNS), responsible for cellular damages [64] and the promotion of tumor growth by angiogenetic processes [65]. This occurs in many types of cancer [66], including gliomas [67,68,69]. Other studies report anti-tumor effects of NO, linked to the amount released, the tumorigenesis phase, and the NO-releasing cells [70].

Microglia/macrophages-derived m/lEVs transport a cargo such as that of donor cells in terms of pro- or anti-inflammatory genes and are capable to modulate microglia phenotype [12,71,72,73]. In our experiments, sEVs include pro- or anti-inflammatory genes of microglia donor cells, but the anti-tumor effect of these vesicles is not mediated by this kind of cargo. Probably, because of that, the effect of sEVs is not mediated by modifications on microglia/macrophage inflammatory status. This result was confirmed not only in CD11b+ cells isolated from glioma-bearing mice (i.e., all tumor-associated microglia/macrophages cells), but also in vitro, on pure microglia. Of note, although arginase 1 gene expression was significantly reduced in CD11b+ cells isolated from glioma-bearing mice upon sEVs treatment, the result was not confirmed on IL4-stimulated microglial cells or by the release of NO by BV2 cells, suggesting the need of further investigations. A previous study on microglia-derived Exos reported their ability to affect microglia itself, decreasing the expression of anti-inflammatory markers: the effect was not ascribed to Exo cargos, but to vesicle engineering with Tetraspanin2 (Tspan2) [73]. Further experiments will help to understand whether the effects of sEVs are region-specific, as recently it was demonstrated that the phenotype of TAMs changes as a function of their position in the tumor mass [74,75].

Many studies focused on the neuronal activity of microglia-derived Exos, and an effect on neuropeptide degradation was shown, directly affecting neurons [76]. The same has been described for astrocytes-derived Exos, which protect neurons against toxic insults, transporting neuroprotective molecules such as neuroglobin, miR190b, or miR34c [77,78]. The anti-tumoral activity of stem cell-derived EVs has been demonstrated in many types of tumors, such as breast cancer [79], fibrosarcoma, and leukemia [80,81].

On the other side, Exos can expand neuroinflammation through inflammasome protein, the nucleotide-binding domain, and leucine-rich repeat containing 1 (NLRP1) in traumatic brain-injured patients [82], spread the β-amyloid peptides in Alzheimer disease patients [10] and propagate the protein TDP-43, of which the aggregation causes frontotemporal dementia [83].

In tumors, sEVs derived from type 2 macrophages promote breast cancer cells invasion [84], and sEVs released by glioma contribute to the acquisition of a tumor-supporting phenotype of infiltrating macrophage/microglia cells [85].

Although it is well established that Exos play a role in the physiopathology of the CNS, their effect is not unique but, as expected, depends on the type of donor cell, the target cell, and the microenvironment in which they are released. In the context of brain tumors, many reports highlight the importance of EVs as modulators of immune response and as a natural vehicle for drug delivery (rev. in [86]).

The potential application of microglia-derived sEVs in glioma is also supported by the observations that they are non-viable materials, can escape the clearance by the immune system, remain stable ex vivo for months and are easy to be produced and stored on a large scale [87]. In addition, EVs can be uptaken by the same target cells with different pathways, such as clathrin-mediated endocytosis and micropinocytosis [88,89,90,91,92]. Our TEM results clearly showed that the membrane of glioma cells merge and internalize EVs into the cytoplasm thorough multiple pathways, with a preference for macropinocytosis and caveolae-mediated endocytosis. It has been shown that sEVs in the cytoplasmatic matrix of glioma cells can escape from endo-lysosome degradation [93], but further studies are needed to clarify this specific feature.

The involvement of miR-124 as a key player in the anti-tumoral effect exerted by BV2-derived sEVs has been suggested by its relative high abundance in the CNS [94,95], and it is a tumor-suppressor microRNA in many tumors [33,34]. Particularly, it is downregulated in GBM samples with respect to healthy brain [90], and its downregulation is related to glioma malignancy [96] and is predictive of poor prognosis in GBM patients [90]. In accordance with our findings, miR-124 inhibits GBM cell proliferation and angiogenesis in gliomas [36,97,98]. In addition, miR-124 overexpression increases survival in a xenogeneic orthotopic intracranial mouse model [36]. As concerns the migration of tumor cells, although it has been demonstrated that the overexpression of miR-124 in human GBM cells (U87MG and U251) induced a lower migratory capability in vitro in the wound-healing assay [96,98], our previous and current results demonstrated that sEVs did not affect GL261 glioma cells in the wound-healing assay [12] and in vivo experiments. The use of the entire cargo of microglia-derived sEVs versus miR-124 as well as the species of cells used could explain these discrepancies that require more detailed studies.

It is important to note that unstimulated microglia express more miR-124 than microglia in co-culture with astrocytes or neurons [99], and in addition to our findings in which the inflammatory state of the donor cell does not affect the antitumor activity of sEVs (Figure 1), this further supports our choice of using sEVs derived from unstimulated microglia for the treatment of glioma-bearing mice. Of note, the lack of effects of the microglia-derived sEVs on microglia phenotype is in line with the observation that in pathological conditions, miR-124 determines a switch of microglia towards an anti-inflammatory phenotype and its inhibition favors a pro-inflammatory phenotype [100,101,102,103]. The partial effect observed by sEVs released by BV2 transfected with the miR-124 inhibitor may be due to other components of vesicle cargos. For example, the aminopeptidase CD13 contained in microglia-derived cargo controls the catabolism of neuropeptides when transported in neurons via Exos [76].

In conclusion, this study suggests that microglia-derived sEVs contribute to maintain brain homeostasis through Glu balance in the extracellular space transporting at least one key regulator of this mechanism, miR-124. In the tumor milieu, an increase of microglia-derived sEVs in glioma-bearing mice exerts an antitumor effect through the regulation of Glu release/uptake from both tumor cells and astrocytes. The results outline a possible mechanism of restoring the homeostatic defensive capacity of the brain against tumor attacks.

## Figures and Tables

**Table 1:** Primer sequences. Forward and reverse primer sequences used for gene expression analysis by qPCR are listed.

Gene	Forward	Reverse
*Arg1*	CTCCAAGCCAAAGTCCTTAGAG	AGGAGCTGTCATTAGGGACATC
*Cd163*	GCTAGACGAAGTCATCTGCACTGGG	TCAGCCTCAGAGACATGAACTCGG
*Cd206*	CAAGGAAGGTTGGCATTTGT	CCTTTCAGTCCTTTGCAAGT
*Fizz1*	CCAATCCAGCTAACTATCCCTCC	ACCCAGTAGCAGTCATCCCA
*Gapdh*	TCGTCCCGTAGACAAAATGG	TTGAGGTCAATGAAGGGGTC
*Il1β*	CAACCAACAAGTGATATTCTCCATG	GATCCACACTCTCCAGCTGCA
*Slc1a2*	CTGGTGCAAGCCTGTTTCC	GCCTGTTCACCCATCTTCC
*Slc1a3*	AGCAGGGAGTCCGTAAACG	AGCATTCCGAAACAGGTAACTTT
*Sc7a11*	CCTCTATTCGGACCCATTTAG	CTGGGTTTCTTGTCCCATATA
*Slc16a1*	GCTTGGTGACCATTGTTGGAAT	CCCAGTCGTGTATTTGTAGTCTCCAT
*Tnf-α*	GTGGAACTGGCAGAAGAG	CCATAGAACTGATGAGAGG
*Ym1*	CAGGTCTGGCAATTCTTCTGAA	GTCTTGCTCATGTGTGTAAGTGA

## Supplementary Materials

The following are available online at https://www.mdpi.com/article/10.3390/cells10082066/s1, Figure S1: Expression analysis of proinflammatory/anti-tumoral-related genes in microglia-derived sEVs and anti-inflammatory/pro-tumoral genes in sEVs-treated microglia, Figure S2: Microglia-derived sEV effects on glioma cell proliferation. Figure S3: Tumor visualization of GL261-RFP-bearing mice and Glast expression on tumor area of GL261-RFP-bearing mice. Table S1: Expression analysis of inflammation-related genes on sEVs derived from microglia.
