# Supplementary material for: Microglia-Derived Small Extracellular Vesicles Reduce Glioma Growth by Modifying Tumor Cell Metabolism and Enhancing Glutamate Clearance through miR-124
Source: Cells. 2021 Aug 12;10(8):2066. doi: 10.3390/cells10082066 (PMC8393731; doi:10.3390/cells10082066)
Supplement: Supplementary file 1 [file cells-10-02066-s001.zip › cells-1292535-supplementary.pdf]

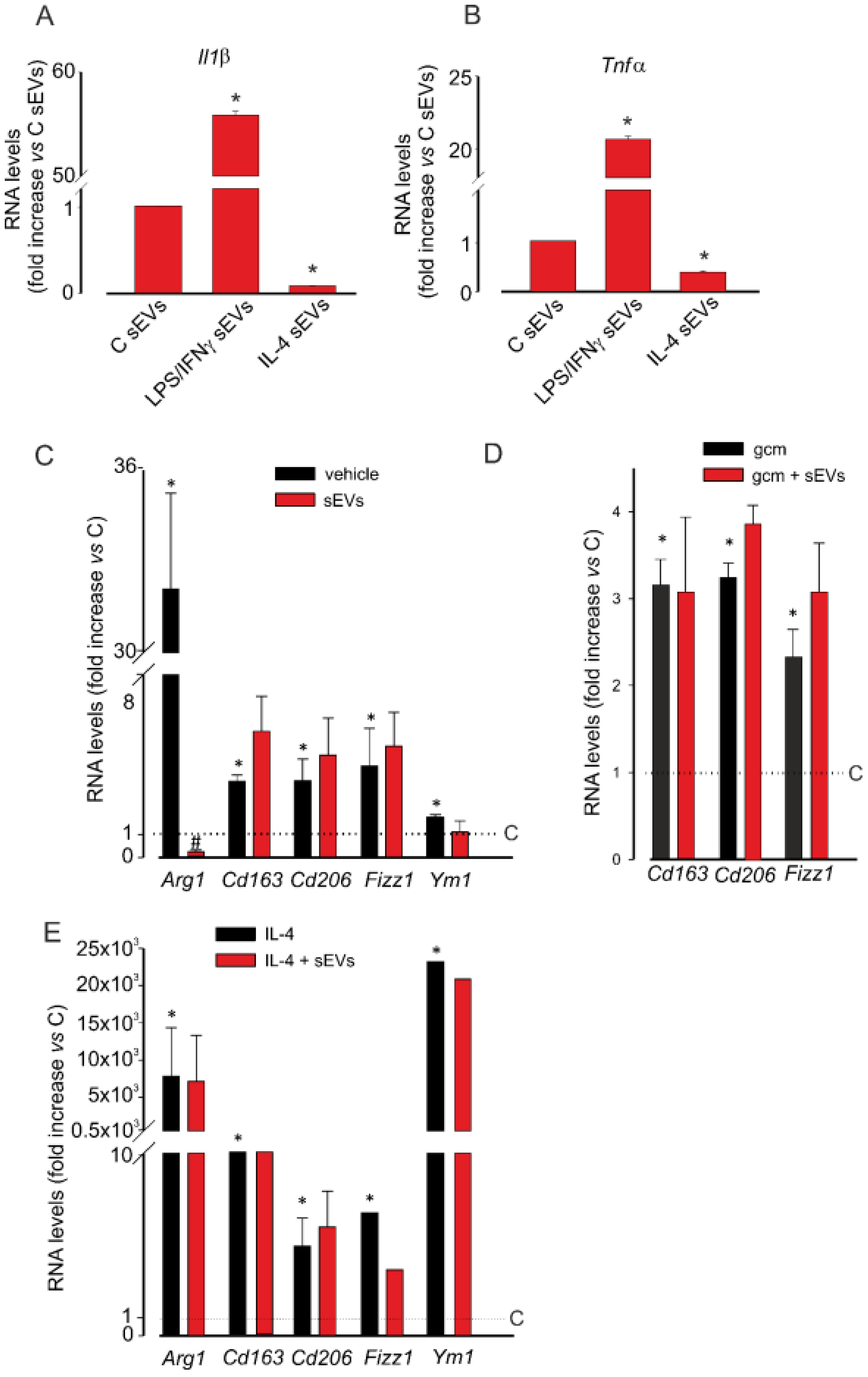

A
B
Il1β
Tnfα
RNA levels (fold increase vs C sEVs)
C sEVs
LPS/IFNγ sEVs
IL-4 sEVs
C
D
vehicle
sEVs
gcm
gcm + sEVs
RNA levels (fold increase vs C)
Arg1
Cd163
Cd206
Fizz1
Ym1
E
IL-4
IL-4 + sEVs

Figure S1: E**xpression analysis of pro-inflammatory/anti-tumoral-related genes in microglia-derived sEVs and anti-inflammatory/pro-tumoral genes in sEVs-treated microglia.** (**A-B**) qPCR of I*l1-b* and *Tnf*α genes expressed in sEVs derived from untreated (C) or INFγ/LPS- or IL4-treated primary microglial cells. Data are the mean ± SE of fold increase *vs* C, normalized on *Gapdh* (used as housekeeping gene), N=3, * p < 0.05 *vs* C, Student's t-test. (**C**) Pro-tumoral anti-inflammatory related genes (*Arg1, Cd163, Cd206,* Y*m1* and *Fizz1*) in CD11b^{+}cells extracted from brains of GL261-bearing mice treated with vehicle or BV2-derived sEVs. Data are the mean ± SE of fold increase respect to contralateral cerebral hemispheres of each animal (C) and all genes expression was normalized vs *Gapdh*, N = 5, * p <0.05 *vs* C, # p <0.05 vs vehicle treated mice, Student's *t*-test. (**D**) Pro-tumoral anti-inflammatory related genes (*Cd163, Cd206,* and *Fizz1*) in primary microglial cells exposed to unconditioned (C) or GL261 conditioned medium (gcm), upon BV2-derived sEVs treatment. Data are the mean ± SE of fold increase respect to (C) and all genes expression was normalized vs *Gapdh*, N = 3, * p <0.05 *vs* C, Student's *t*-test. (**E**) Pro-tumoral anti-inflammatory related genes (*Arg1, Cd163, Cd206, Ym1* and *Fizz1*) in primary microglial cells untreated (C), exposed to IL4 or IL4 plus BV2-derived sEVs. Data are the mean ± SE of fold increase respect to (C) and all genes expression was normalized vs *Gapdh*, N = 3, * p <0.05 *vs* C, Student's *t*-test.

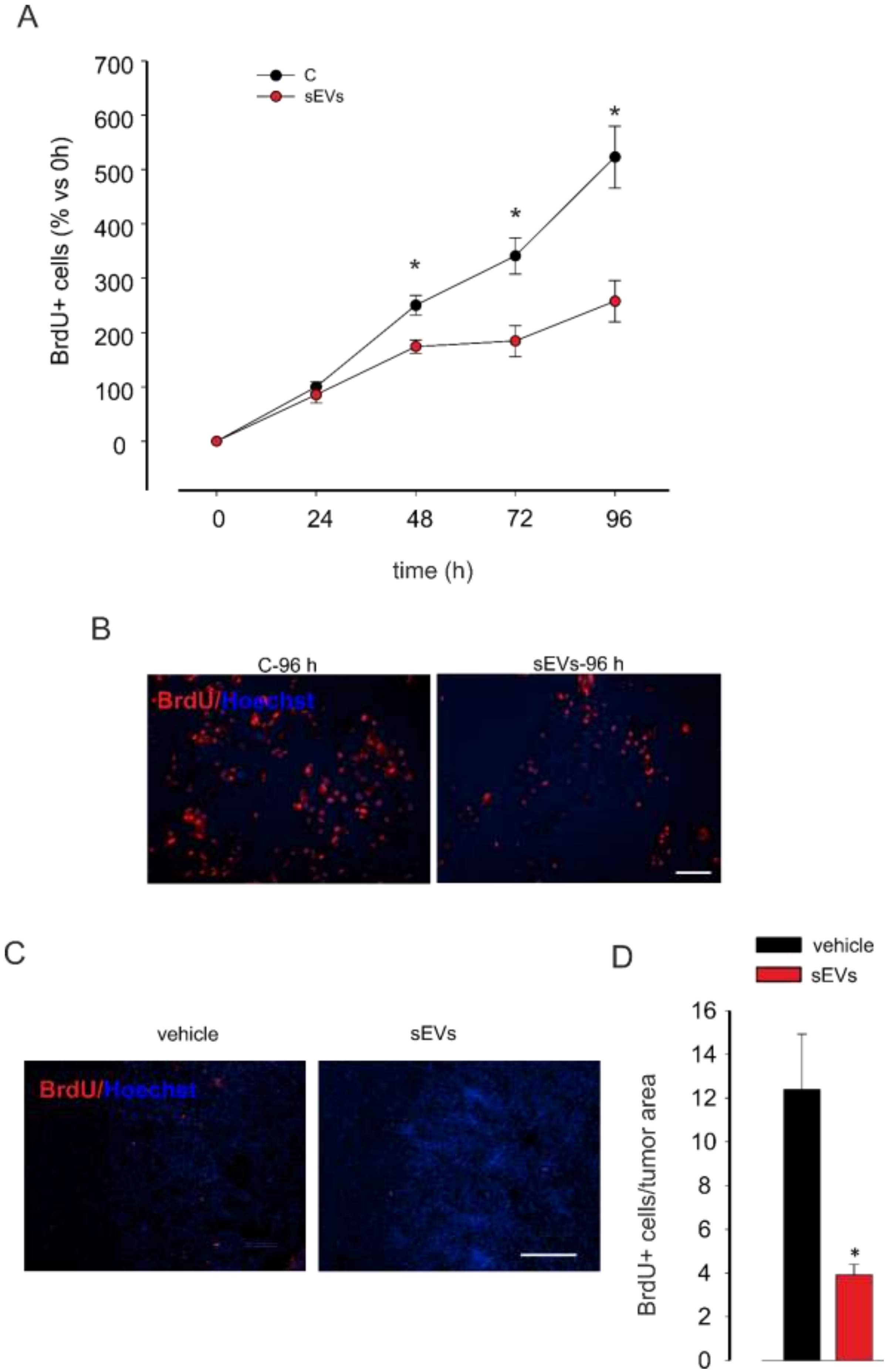
A
700
600
500
400
300
200
100
0
BrdU+ cells (% vs 0h)
C
sEVs
0
24
48
72
96
time (h)
B
C-96 h
sEVs-96 h
BrdU/Hoechst
C
vehicle
sEVs
BrdU/Hoechst
D
vehicle
sEVs
16
14
12
10
8
6
4
2
0
BrdU+ cells/tumor area

Figure S2: Microglia-derived sEV effects on glioma cell proliferation. (A) *In vitro* cell proliferation measured as immunofluorescence analysis of $BrdU^+$ GL261 untreated (C) or treated with BV2-derived sEVs for 0, 24, 48, 72 and 96 h. Data are reported as % ± SE, N = 3, * $p < 0.05$ *vs* time 0 (0 h), Student's *t*-test. (**B)** Representative immunofluorescence of time point 96 h of (A), BrdU in red, Hoechst in blue, scale bars, 100 µm. **(C)** *In vivo* glioma cell proliferation measured as immunofluorescence analysis of $BrdU^+$ cells in the tumor core of coronal brain sections of GL261-bearing mice vehicle treated (on the left) or treated with BV2-derived sEVs (on the right); BrdU in red, Hoechst in blue, scale bars, 20 µm. (D) Quantification of (C), data are expressed as the signal coverage area of BrdU normalized on tumor area ($BrdU^+$/tumor area) ± SE, N = 3, * $p < 0.05$ *vs* vehicle, Student's *t*-test.

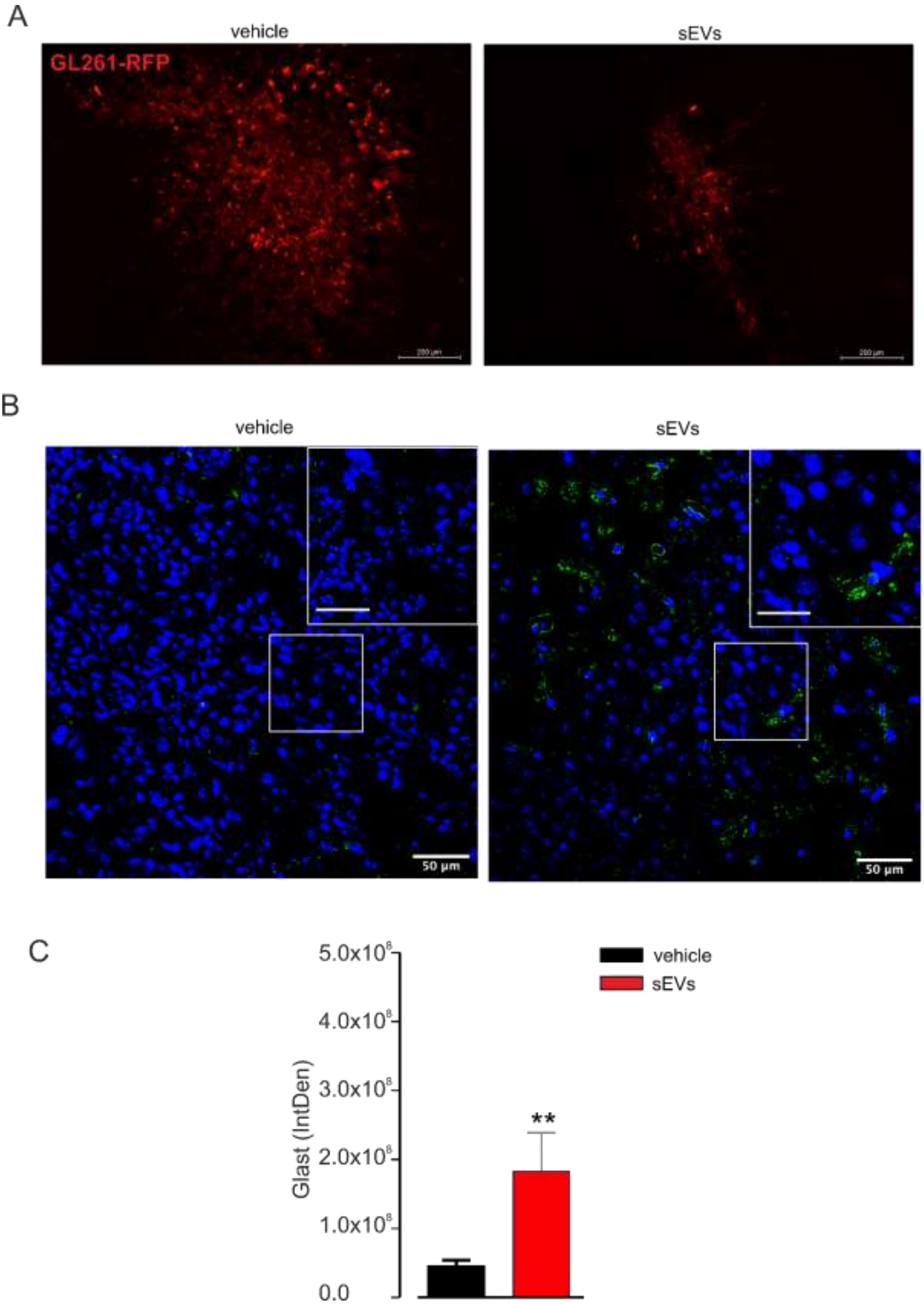

Figure S3: (A) Tumor visualization of GL261-RFP-bearing mice. Representative tumor area (GL261 in red) of coronal brain sections of GL261-bearing mice vehicle-treated (on the left) or treated with BV2-derived sEVs (on the right), scale bar, 200 µm. **B) Glast expression in tumor area of GL261-bearing mice.** Representative immunofluorescence analysis for Glast (in green, Hoechst in blue) in the tumor core of coronal brain sections of GL261-bearing mice vehicle-treated (on the left) or treated with BV2-derived sEVs (on the right), scale bars, 50 µm. In the inset an enlargement is shown, scale

bars, 25 µm. **(C)** Quantification of (B), data are expressed as IntDen mean ± SE, N = 3, ** *p* ≤ 0.001 *vs* vehicle, Student's *t*-test.

Table S1: Expression analysis of inflammation related genes on sEVs derived from microglia. mRNAs isolated from sEVs released by untreated microglia (control) or microglia treated with LPS/IFNg or microglia treated with IL4 were analysed by NanoString., N = 4, data are expressed as fold increase respect to *gapdh* used as housekeeping gene, * *p* <0.05 *vs* control, Mann-Whitney Rank Sum test.

Gene	Control	LPS/IFNγ	IL4
Ccl2	5,99 ± 2,5	128,08 ± 26,9 *	7,8 ± 2,5
Ccl3	29,93 ± 16,34	441,21 ± 72,5	10,74 ± 3,6
Ccl5	66,12 ± 36,3	5938,56 ± 1612,4*	50,09 ± 10,8
Ccl7	1,31 ± 0,2	108,50 ± 10,2*	6,33 ± 1,9
Cebpb	11,19 ± 5,5	118,94 ± 15,4*	15,14 ± 5,2
Cxcl9	1,31 ± 0,2	476,99 ± 186,5*	1,06 ± 0,2
Hif1a	22,29 ± 11,9	214,48 ± 18,2*	21,02 ± 7,4
Il1b	12,6 ± 6,32	703,55 ± 239*	1,06 ± 0,2*
Ptgs2	1,31 ± 0,2	779,24 ± 354,34*	1,06 ± 0,1
Rapgef2	15,35 ± 7,9	72,50 ± 10,1*	5,6 ± 1,2*
Tnfa	12,69 ± 5,5	262,12 ± 56,19*	4,86 ± 1,0*
